# Growth-Associated Protein-43 Loss Promotes Ca^2+^ and ROS Imbalance in Cardiomyocytes

**DOI:** 10.3390/antiox14030361

**Published:** 2025-03-19

**Authors:** Michele Bevere, Caterina Morabito, Delia Verucci, Noemi Di Sinno, Maria A. Mariggiò, Simone Guarnieri

**Affiliations:** 1 Department of Neuroscience, Imaging and Clinical Sciences and Center for Advanced Studies and Technology (CAST), University “G. d’Annunzio” of Chieti-Pescara, 66100 Chieti, Italy; michele.bevere@univr.it (M.B.); caterina.morabito@unich.it (C.M.); delia.verucci@studenti.unich.it (D.V.); disinnon@gmail.com (N.D.S.); maria.mariggio@unich.it (M.A.M.); 2 ARC-Net Applied Research on Cancer Centre, University and Hospital Trust of Verona, 37134 Verona, Italy

**Keywords:** GAP-43, Ca^2+^, calmodulin, heart, ROS, cardiomyocytes, mitochondria

## Abstract

Growth-Associated Protein-43 (GAP-43) is a calmodulin-binding protein, originally found in neurons, that in skeletal muscle regulates the handling of intracellular Ca^2+^ dynamics. According to its role in Ca^2+^ regulation, myotubes from GAP-43 knockout (GAP-43^−/−^) mice display alterations in spontaneous Ca^2+^ oscillations and increased Ca^2+^ release. The emerging hypothesis is that GAP-43 regulates CaM interactions with RyR and DHPR Ca^2+^ channels. The loss of GAP-43 promotes cardiac hypertrophy in newborn GAP-43^−/−^ mice, extending the physiological role of GAP-43 in cardiac muscle. We investigated the role of GAP-43 in cardiomyocytes derived from the hearts of GAP-43^−/−^ mice, evaluating intracellular Ca^2+^ variations and the correlation with the levels of reactive oxygen species (ROS), considering their importance in cardiovascular physiology. In GAP-43^−/−^ cardiomyocytes, we found the increased expression of markers of cardiac hypertrophy, Ca^2+^ alterations, and high mitochondria ROS levels (O_2_^•−^) together with increased oxidized functional proteins. Treatment with a CaM inhibitor (W7) restored Ca^2+^ and ROS alterations, possibly due to high mitochondrial Ca^2+^ entry by a mitochondrial Ca^2+^ uniporter. Indeed, Ru360 was able to abolish O_2_^•−^ mitochondrial production. Our results suggest that GAP-43 has a key role in the regulation of Ca^2+^ and ROS homeostasis, alterations to which could trigger heart disease.

## 1. Introduction

Growth-Associated Protein-43 (GAP-43) is highly expressed during axon growth and synaptogenesis. Its involvement in neuronal development has been demonstrated by gene knockout studies. Homozygous GAP-43 knockout (GAP-43^−/−^) mice are characterized by high neonatal lethality, resulting in 5–10% survival to adulthood [1]. Although GAP-43 has long been classified as a neuron-specific protein, there are several studies that describe the presence of this protein in non-nervous tissues, as well [2,3]. In skeletal muscle, it has been found in meromyosin-positive cells in the limbs of chicken embryos and in human skeletal muscle in subjects suffering from interstitial myositis, suggesting its involvement in the regenerative processes associated with muscle diseases [4,5]. In mouse skeletal muscle, the expression and intracellular localization of GAP-43 follow a distinct progression associated with differentiation from myoblasts to myotubes. Undifferentiated cells show a robust nuclear localization of GAP-43 with an irregular pattern of diffuse spots in the cytosol. In myotubes, the protein is re-localized at cytoplasmic level, forming regular transversal streaks. Co-localization and functional analyzes demonstrated that the protein is positioned close to Ca^2+^ release units and the mitochondria, suggesting that GAP-43 may play a key role in Ca^2+^ homeostasis in skeletal muscle [6].

In addition, interestingly, in the skeletal muscle of lower vertebrates (amphibians and fishes), GAP-43 localizes closely to the triad junction, suggesting a conserved physiological role across species [7]. Depending on the species, GAP-43 is composed of 194–238 amino acids, containing a conserved IQ motif, which makes the protein capable of binding calmodulin (CaM). Binding to CaM is conditioned by the presence of high intracellular Ca^2+^ concentrations or by the phosphorylation of a serine residue (ser41) within the CaM-binding IQ domain. In these conditions, the binding affinity between CaM and GAP-43 is drastically reduced, allowing CaM mobilization towards its intracellular targets [8]. In this regard, intracellular Ca^2+^ homeostasis was investigated in myotubes derived from differentiated satellite cells of wild-type (WT) and GAP-43^−/−^ mice. These findings revealed an increased amplitude and frequency of spontaneous and stimulated Ca^2+^ oscillations due to increased Ca^2+^ currents via dihydropyridine and ryanodine receptor Ca^2+^ channels (DHPR and RyR, respectively) as a consequence of an alteration in CaM-operated control. The proposed hypothesis was that GAP-43 regulated downstream CaM interactions with RyR and DHPR to modulate Ca^2+^ channel opening. Indeed, W7, a specific CaM inhibitor, was able to restore the Ca^2+^ currents in GAP-43^−/−^ myotubes. It has been postulated that GAP-43 provides a “functional microdomain” that locates the CaM near the Ca^2+^ release units [9].

Studies carried out in adult GAP-43^−/−^ mice revealed a reduced expression of force, which was accompanied by a reduced body weight. Ultra-structural analyses by electron microscopy on the diaphragm and EDL muscles (early and late functional maturing tissues, respectively) of GAP-43^−/−^ mice have shown a delay in the degree of triad maturation, as well as a reduction in their number, even if the formation of the neuromuscular junctions was normal. These data highlighted that GAP-43 may play a role in the processes that accompany the development and functional maturation of skeletal muscle [9]. Interestingly, Rahmati and Taherabadin found lower levels of GAP-43 in atrophied gastrocnemius muscle in diabetic animals, suggesting that the level of GAP-43 could be a critical factor in skeletal muscle mass and size [10].

Recently, published data from our laboratory demonstrated that GAP-43 is expressed also in mouse heart muscle and that its expression is related to tissue development: at birth, the protein is expressed at high levels, which decrease towards adulthood. In addition, we have shown that GAP-43 is located near dyads. Interestingly, GAP-43^−/−^ mice develop cardiac remodeling and hypertrophy, showing an increased cardiac mass index and a thicker ventricular wall and interventricular septum, with a reduced ventricular chamber area. Moreover, cross-sectional areas of GAP-43^−/−^ heart fibers are increased, as well as the expression levels of myosin heavy chain. These results emphasize that cardiac hypertrophy could be at least a co-morbidity factor in sudden death in the offspring of GAP-43^−/−^ mice [11].

Also, the dyshomeostasis in Ca^2+^ handling is often reported in hypertrophic cardiomyocytes in which the prolonged activation of Ca^2+^-calmodulin-dependent protein kinase II (CaMKII) signals is involved, playing a key role [12]. In this regard, the functional link between intracellular Ca^2+^ control and mitochondrial redox status is of fundamental importance. Indeed, under physiological conditions, reactive oxygen species (ROS) fluctuations act as essential signaling to control cellular functions in the cardiovascular system [13,14].

The aim of this study is to define the role of GAP-43 in intracellular Ca^2+^ homeostasis in cardiomyocytes and its relationship with ROS balance.

Using cardiomyocytes isolated from the hearts of neonatal WT and GAP-43^−/−^ C57BL/6 mice, intracellular Ca^2+^ and ROS levels were analyzed. GAP-43^−/−^ cardiomyocytes showed altered spontaneous Ca^2+^ oscillations and higher ROS levels compared to WT cardiomyocytes.

Our findings suggest that GAP-43 has a role in controlling intracellular Ca^2+^ homeostasis and balancing mitochondrial ROS levels, alterations to which may be responsible for the initiation of a hypertrophy program in cardiac cells.

## 2. Materials and Methods

### 2.1. Chemicals and Materials

Unless otherwise indicated, cell culture media, sera, antibiotics, and cell culture dishware were obtained from Thermo Fisher Scientific (Monza, Italy), and reagents and standards were obtained from Merck Life Science S.r.l. (Milan, Italy).

### 2.2. Animal Models

C57BL/6 GAP-43 heterozygous (GAP-43^+/−^) mice were kindly provided by Karina F. Meiri, State University of New York, NY, USA. GAP-43^+/−^ mice were crossed, and progeny was genotyped by PCR to discriminate WT and homozygous GAP-43^−/−^ mice from GAP-43^+/−^ ones, according to the protocol described by Bevere et al., 2022 [11]. All experiments were performed using WT and GAP-43^−/−^ mice.

The experimentation on, and housing of, animals were performed at the Center for Advanced Studies and Technology (CAST), Chieti, in accordance with European Guidelines for the use of animals in research (2010/63/EU) and the requirements of Italian laws (D.L. 26/2014) and were approved by the “Organismo preposto al benessere animale” (OpBA) of the “G. d’Annunzio” University of Chieti-Pescara and with authorization no. 4739.n.ias from the Italian Ministry of Health [principal investigator, S.G.].

### 2.3. Isolation of Primary Cardiomyocytes from Neonatal Mouse Heart

After genotyping screening, WT and GAP43^−/−^ neonatal mice were sacrificed 24h after their birth according to the authorized procedure, and their hearts were removed to obtain primary cardiomyocytes using the Thermo Fisher Pierce™ Primary Cardiomyocyte Isolation Kit (cat. n. 88281, Thermo Fisher Scientific). Isolated hearts were washed twice with ice-cold Hanks’ Balanced Salt Solution without Ca^2+^/Mg^2+^ (HBSS) to remove blood from tissues then minced into smaller pieces and, immediately, washed twice in ice-cold HBSS. The tissue was subjected to chemical digestion using Cardiomyocyte Isolation Enzyme 1 (papain) and Cardiomyocyte Isolation Enzyme 2 (thermolysin) in a 37 °C incubator for 35 min. The enzyme solution was removed, and the samples were washed twice with ice-cold HBSS. DMEM from Primary Cell Isolation kit (supplemented with 5% (*v*/*v*) FBS and Penicillin/Streptomycin, EuroClone, Pero, Italy) was added, and samples were mechanically resuspended and dissociated by pipetting. The cell number was determined, and cardiomyocytes (1,200,000–1,400,000 cells) were plated in precoated collagen 35 mm dishes. Cells were maintained in the DMEM from Primary Cell Isolation kit for 24 h from isolation, then the medium was replaced with DMEM from Primary Cell Isolation kit, supplemented with growth supplement to support cardiomyocyte growth and inhibit fibroblast proliferation. The medium was changed on the fourth day, and, after the seventh day, cultures were used for the experiments. GAP43 expression was tested in WT and GAP43^−/−^ heart-derived cultures (see Figure S1).

### 2.4. Western Blotting Assays

The protein extracts used for Western blotting were from cardiomyocytes isolated from WT or GAP-43^−/−^ neonatal mouse hearts. The cardiomyocytes were washed in cold PBS, scraped, and collected in ice-cold RIPA lysis buffer (Thermo Fisher Scientific). After centrifugation (10,000× *g*, for 10 min at 4 °C), the protein concentrations in the supernatants were assayed (Bio-Rad protein assay; Bio-Rad Laboratories S.r.l, Segrate, Italy). Protein extracts (20 μg) were resuspended in Laemmli buffer and separated by SDS-PAGE on 8%, 10%, or 12% (*w*/*v*) homogeneous slab gels and then electroblotted onto nitrocellulose membranes (AmershamTM–ProtranTM–0.45 μm NC, GE Healthcare, Cologno Monzese, Italy). The membranes were blocked with a TBS-Tween 0.1% solution with 5% milk and then incubated overnight at 4 °C with the following primary antibodies: mouse monoclonal antibody anti-cardiac myosin heavy chain (cMYH, 1:1000 dilution; Thermo Fisher Scientific); mouse monoclonal antibody anti α-actinin (1:1000 dilution; Merck Life Science S.r.l., Bucharest, Romania); mouse monoclonal antibody anti-MYH7 (1:1000 dilution; Santa Cruz Biotechnology Inc., Santa Cruz, CA, USA); rabbit monoclonal anti-CaMKII phosphorylated (p-CaMKII, 1:1000 dilution; Cell Signaling Technology, Danvers, MA, USA); rabbit monoclonal anti-CaMKII (1:1000 dilution; Cell Signaling Technology, Danvers, MA, USA); mouse monoclonal anti-Nkx-2.5 (1:1000 dilution; Santa Cruz Biotechnology Inc.); rabbit polyclonal anti-GATA4 (1:1000 dilution; Merck Life Science S.r.l.); mouse monoclonal antibody anti-Cav1.2 (1:1000 dilution; Thermo Fisher Scientific); rabbit monoclonal antibody anti-RyR2 (1:1000 dilution; Thermo Fisher Scientific); rabbit polyclonal antibody anti-calcineurin (1:500 dilution; Merck Life Science S.r.l.); rabbit polyclonal antibody anti-SERCA2 (1:1000 dilution; Thermo Fisher Scientific); rabbit polyclonal antibody anti-SOD1 (1:1000 dilution; Thermo Fisher Scientific); mouse monoclonal antibody anti-SOD2 (1:1000 dilution; Thermo Fisher Scientific); rabbit polyclonal antibody anti-catalase (1:1000 dilution; Thermo Fisher Scientific); rabbit polyclonal anti-GPX1 (1:1000 dilution, Thermo Fisher Scientific); mouse monoclonal antibody anti-NOX2 (1:500 dilution; Santa Cruz Biotechnology Inc.); rabbit polyclonal anti-nitrotyrosine (1:1000 dilution; Merck Life Science S.r.l.); mouse monoclonal anti-4-Hydroxynonenal (4-HNE, 1:1000 dilution; Thermo Fisher Scientific); rabbit polyclonal antibody anti-pRyR2 (1:1000 dilution; Thermo Fisher Scientific); rabbit polyclonal antibody anti-p-PLB (Ser16, Thr17) (1:1000 dilution; Thermo Fisher Scientific). After washing, the membranes were incubated with horseradish-peroxidase-conjugated appropriate secondary antibodies (1:10,000 dilution) for 1 h at room temperature (RT), and the signals were detected using chemiluminescence kits (GE Healthcare) and an image acquisition system (Uvitec, Cambridge, UK). A mouse monoclonal antibody anti-β-actin (1:1000 dilution; Santa Cruz Biotechnology Inc.), a rabbit monoclonal antibody anti-vinculin (1:10,000 dilution Abcam, Cambridge, UK), or a mouse monoclonal antibody anti-GAPDH (1:10,000 dilution; Merck Life Science S.r.l.) were used as loading controls. Where indicated, the blots were stripped according to the manufacturer’s protocol for the stripping solution (Thermo Fisher Scientific) before re-probing.

### 2.5. Cytosolic and Mitochondrial Ca^2+^ Imaging

Intracellular Ca^2+^ levels were monitored using Fluo-4 acetoxymethyl ester (Fluo-4/AM, Thermo Fisher Scientific). An upright Zeiss Axio Examiner microscope (Carl Zeiss, Jena, Germany) was used, equipped with a 40× water immersion objective (0.75 NA) and connected by an optical fiber to a 75 W Xenon lamp and a monochromator (OptoScan; Cairn Instrument, Instrments Faversham, Kent, UK). The WT and GAP-43^−/−^ cardiomyocytes were incubated with 5 µM Fluo-4/AM in normal external solution (NES: 140 mM NaCl, 2.8 mM KCl, 2 mM CaCl_2_, 2 mM MgCl_2_, 10 mM glucose, 10 mM Hepes, pH 7.3) supplemented with 1% (*w*/*v*) bovine serum albumin (BSA) for 30 min at 37 °C. Recordings on Fluo4-loaded cells were performed in NES or, where indicated, in NES containing 1 mM n-acetyl-l-cysteine (NAC, after a pre-incubation of 24 h in the cell growth medium containing 1 mM NAC) or in NES containing 20 μM n-(6-aminohexyl)-5-chloro-1-naphthalenesulfonamide hydrochloride (W7, after a pre-incubation of 10 min in cell growth medium containing 20 μM W7). The Fluo4-loaded cells were excited at 488 nm, and the fluorescence images were acquired for 60 s at 1 frame/50 ms with a 12-bit digital EMCCD camera (PhotoEvolve 512; Photometrics; Tucson, AZ, USA). The temporal analysis was calculated as f/f0, where f is the mean fluorescence intensity signal of a selected cell area of a single loaded cell acquired during a time lapse, and f0 is the mean fluorescence intensity of the same cell calculated from the first time point acquired. Each Ca^2+^ trace was analyzed using AnomalyExplorer software (ver. 1.0) to identify normal and abnormal Ca^2+^ transients [15]. The abnormal signals were categorized into different subgroups based on the abnormalities recognized by the software as follows: low peaks, double peaks, middle picks, irregular phases, and oscillations. According to the software user interface, the following user-defined setting was used for all conditions analyzed: low peaks (10–40%); double peaks (20–28%); medium peaks (0–38%); irregular phases (100%); oscillations (31%).

The total number of subgroup-specific abnormalities was quantified from the Ca^2+^ signals for each condition analyzed (for details, see also Supplementary Method S1).

To evaluate mitochondrial Ca^2+^ levels, WT and GAP-43^−/−^ cardiomyocytes were simultaneously incubated with 5 μM Fluo-4 AM and 5 μM MitoTracker Deep Red (Thermo Fisher Scientific) in NES supplemented with 1% BSA for 45 min at RT in the dark. After the incubation time, recordings on cardiomyocytes were performed with a confocal microscope (Zeiss LSM 800) equipped with Zeiss Axiovert 200 inverted microscope, a Plan Neofluar oil-immersion objective (40×/1.3 NA), and the LSM 3.0 image analysis software (Carl Zeiss). The fluorescence signals were simultaneously acquired by setting the excitation at 488 nm for Fluo-4 and 640 nm for MitoTracker Deep Red. Image acquisition was performed at 2 frames/s for 1 min. The quantitative analysis of fluorescence intensity of the Fluo-4 in the MitoTracker Deep Red-stained mitochondria was determined using Fiji-ImageJ software (ver. 1.54f) (National Institutes of Health, NIH, Bethesda, MD, USA). Fluorescence intensity was quantified using Fiji Image J software. The mitochondrial Ca^2+^ levels were calculated in cell regions in which Fluo-4 and MitoTracker Deep Red signals co-localized. For each cell, the mitochondria Fluo-4 mean fluorescence intensity value, expressed as arbitrary unit (A.U.), and area were calculated; the data were expressed as mean fluorescence per area unit (A.U./μm^2^). Some experiments were performed in the presence of 10 µM Ru360, an inhibitor of mitochondrial Ca^2+^ uniporter (Calbiochem, Merck Life Science S.r.l., Darmstadt, Germany), which was applied 5 min before acquisition and during recordings.

### 2.6. ROS and Mitochondrial Superoxide Anion Level Measurements

ROS or mitochondrial superoxide anion (O_2_^•−^) levels were evaluated using confocal microscopy (Zeiss LSM 800) and specific dyes: 2′,7′-dichlorodihydrofluorescein diacetate (H2DCF-DA, Thermo Fisher Scientific) for ROS levels and MitoSOX RED (Thermo Fisher Scientific) for O_2_^•−^ levels. WT and GAP-43^−/−^ cardiomyocytes were incubated in NES for 40 min at 37 °C with 10 µM H2DCF-DA or for 15 min at 37 °C with 5 μM MitoSOX. Recordings on H2DCF-DA- or MitoSOX-loaded cells were performed in NES or, where indicated, in NES containing 1 mM NAC (after a pre-incubation of 24 h in cell growth medium containing 1 mM NAC), 20 μM W7 (after a pre-incubation of 10 min in growth medium containing 20 μM W7), or 10 µM Ru360 (after a pre-incubation of 5 min in growth medium containing 10 μM Ru360). Fluorescence signals were acquired at 488 nm for H2DCF-DA or 543 nm for MitoSOX Red. Fluorescence intensity was quantified using Fiji Image J software. The ROS levels were calculated by outlining the regions corresponding to the cell area using ROIs on the acquired fields. For each cell, the mean fluorescence intensity value, expressed as arbitrary unit (A.U.), and area were calculated; the data were expressed as mean fluorescence per area unit (A.U./μm^2^). The O_2_^•−^ levels were expressed as arbitrary unit (A.U.) of fluorescence per area unit (A.U./μm^2^).

### 2.7. Mitochondrial Membrane Potential Measurements

Mitochondrial membrane potentials were determined using JC-1 (5,5′,6,6′-tetrachloro-1,1′,3,3′-tetraethylbenzimidazolylcarbocyanine iodide/chloride; Thermo Fisher Scientific), a cationic carbocyaninic dye that accumulates in the mitochondria. WT and GAP-43^−/−^ cardiomyocytes were incubated for 10 min at 37 °C with 5 μM JC-1 in cell medium. After washing with NES, the loaded cells were observed and analyzed using the same setup and software employed for evaluating intracellular ROS levels. Fluorescence signals were acquired using an excitation of 488 nm and collected at 522 nm for J-monomer (green fluorescence) and 605 nm for J-aggregates (red fluorescence) [16]. Data were expressed as a ratio between red and green fluorescence in JC-1-loaded cells.

### 2.8. Immunofluorescence Staining

WT and GAP-43^−/−^ cardiomyocytes were fixed using 4% paraformaldehyde for 10 min at RT. After three washings with PBS, cardiomyocytes were permeabilized using a 0.2% Triton X-100 solution for 10 min, incubated in a blocking buffer (PBS containing 10% goat serum) for 1 h at RT, and incubated overnight at 4 °C with rabbit monoclonal anti-TOM20 (1:500 dilution; Thermo Fisher Scientific) or with mouse monoclonal antibody anti α-actinin (1:100 dilution; Merck Life Science S.r.l.). Primary antibodies were revealed by 1 h incubation with the appropriate secondary Alexa 488 goat anti-IgG (1:200 dilution; Thermo Fisher Scientific) and the images were acquired using a confocal microscope (Zeiss LSM 800). α-actinin-stained cells were used to quantify cell area using ImageJ software.

### 2.9. Measurements of Glucose and Lactate Levels in Cell Culture Medium

The cell medium of WT or GAP-43^−/−^ cardiomyocytes was removed and centrifuged at 13,000× *g* for 15 min at 4 °C to remove cell debris. Glucose and lactate levels in the supernatants were assayed using a Free Style Optium glucometer (Abbot Laboratories, Rome, Italy) and a Lactate Pro Analyser (Arkray Inc., Kyoto, Japan), respectively. The values were expressed as grams of glucose (g glucose) or moles of lactate (mol lactate) normalized with the protein concentration of the corresponding cell samples [17].

### 2.10. Statistical Analysis

The biological model of this study was represented by primary cell cultures isolated from the hearts of WT or GAP-43^−/−^ mice, and the experimental approaches that we used included analyses of cell populations (i.e., Western blotting or measurements of glucose and lactate levels in the media) or of single cells (i.e., fluorescence live imaging or cell immune-staining), which required the appropriate choice of sample size. We used 3–5 different biological samples (N, i.e., hearts from different animals) to isolate different cardiomyocyte’ populations and to perform 3–6 independent experiments. In the single-cell imaging approach, or cell immunostaining for each biological sample (each isolated cell population), technical replicates were performed by acquiring different randomly selected microscopic fields (from 3 to 15 fields), and the total number of analyzed cells (n) were counted. The related sample size is reported in the figure captions.

The data were expressed as the means ± standard error of the mean (S.E.M.) from at least three biological samples and compared by Student’s *t*-test or ANOVA with post-hoc Tukey’s multiple comparison test, using Prism5 software (GraphPad, San Diego, CA, USA). *p* values < 0.05 were considered statistically significant.

## 3. Results

### 3.1. GAP-43^−/−^ Cardiomyocytes Express a Hypertrophic Phenotype

**Figure 1:**
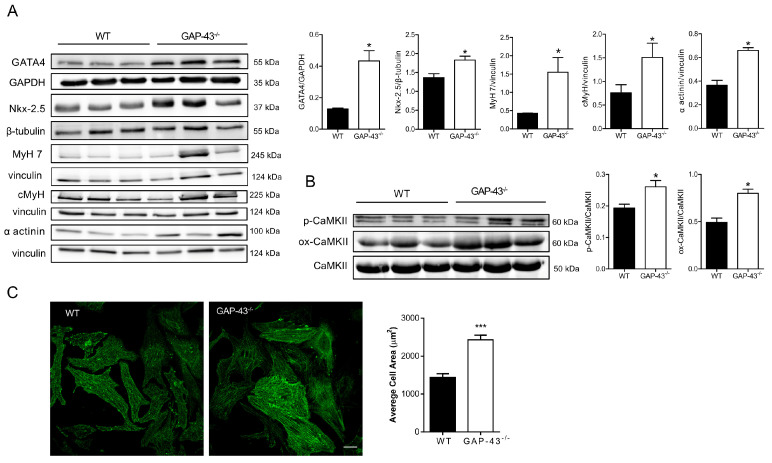
Key factors involved in cardiac hypertrophy. (**A**) Western blots of GATA4, Nkx-2.5, MyH7, cMyH, and α-actinin proteins derived from WT or GAP-43^−/−^ cardiomyocytes, and the corresponding densitometric analyses. (**B**) Western blotting of p-CaMKII and Met281/282 oxidized CaMKII (ox-CaMKII) proteins from WT or GAP-43^−/−^ cardiomyocytes and the corresponding densitometric analyses. The densitometric analyses in (**A**,**B**) are plotted as the ratio between the optical density (OD) × mm^2^ of each band and the OD × mm^2^ of the corresponding loading control (GAPDH, β-tubulin, Vinculin, and CaMKII). (**C**) Representative images of WT and GAP-43^−/−^ cardiomyocytes stained for α-actinin and quantitative analyses of cell area (Bar = 20 µm). Data are expressed as means ± S.E.M. from N = three biological samples in panels (**A**–**C**) with n = 143 WT and 147 GAP-43^−/−^ cardiomyocytes in panel (**C**). * *p* < 0.05; *** *p* < 0.001 (Student’s *t*-test).

Previous studies carried out in our laboratory on newborn GAP-43^−/−^ mice revealed an increase in cardiac size and bilateral ventricular hypertrophy. To evaluate the presence of markers involved in cardiac hypertrophy, cardiomyocytes isolated from neonatal WT and GAP-43^−/−^ hearts were examined after 7 days of culture. Western blot analyses (Figure 1A) revealed increased expression levels of GATA-4 and Nkx2.5, both transcription factors involved in heart development and hypertrophic growth, in GAP-43^−/−^ cardiomyocytes compared to WT ones.

Also, other markers of hypertrophy were found to be increased. Indeed, GAP-43^−/−^ cardiomyocytes showed increased levels of myofibrillar cardiac proteins, such as the myosin heavy chain (MyH) and α-actinin, as well as increased levels of the β isoform of MyH (MyH 7) (Figure 1A). Also, the levels of the phosphorylated and oxidized form of CaMKII were significantly higher in GAP-43^−/−^ cardiomyocytes compared to WT ones (Figure 1B). These post-translational modified forms of CaMKII are involved in the signaling of Ca^2+^-mediated or -regulated activities such as excitation–contraction and excitation–transcription coupling. The presence of a hypertrophic phenotype in GAP-43^−/−^ cardiomyocytes was also confirmed by quantitative analyses of the cell area that resulted in increased GAP-43^−/−^ cardiomyocytes compared to WT ones (Figure 1C).

### 3.2. GAP-43^−/−^ Cardiomyocytes Show Intracellular Ca^2+^ Dyshomeostasis

**Figure 2:**
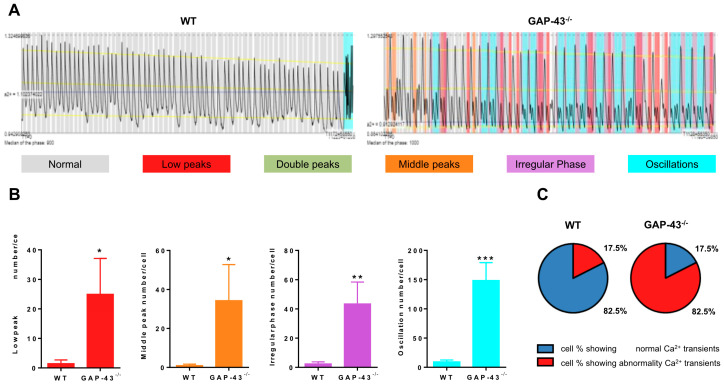
Ca^2+^ transient abnormalities. (**A**) Representative Ca^2+^ signal abnormality patterns detected by AnomalyExplorer software (ver. 1.0) [15] in WT (left image) and GAP-43^−/−^ (right image) cardiomyocytes loaded with Fluo-4. Each color-coded represents both normal Ca^2+^ signals (in gray) and different abnormalities such as low peaks, double peaks, medium peaks, oscillations, and irregular phases. (**B**) Quantitative analysis of the different Ca^2+^ transient abnormalities. The values are means ± S.E.M. from N = three biological samples; a total number of 251 WT and 318 GAP-43^−/−^ cardiomyocytes were examined * *p* < 0.05, ** *p* < 0.01, *** *p* < 0.001 (Students’ *t*-tests). (**C**) Pie charts indicate the cell percentages showing different Ca^2+^ transient signals.

Intracellular Ca^2+^ is a critical regulator of cardiomyocyte function. In heart contraction, it not only acts as a link between electrical signal and mechanical coupling but also controls numerous cardiomyocyte activities, including gene transcription and mitochondrial functions. For this reason, we investigated the spontaneous Ca^2+^ variations in WT and GAP-43^−/−^ cardiomyocytes (Supplemental Materials, Videos S2 and S3, respectively). We used conventional fluorescence video microscopy in living cells loaded with the Ca^2+^ probe Fluo-4 and analyzed the patterns recorded using a software tool (AnomalyExplorer) capable of highlighting anomalies in the spontaneous variations in intracellular Ca^2+^ (Figure 2A; see also Section 2 and Supplementary Method S1).

The results obtained using this approach revealed that a large number of GAP-43^−/−^ cardiomyocytes showed the presence of abnormalities in the spontaneous intracellular Ca^2+^ variations; this number was more than 4-fold higher than the WT one (Figure 2C). Among the anomalies recognized, we found that the presence of low peaks, middle peaks, irregular phases, and oscillations appeared to be significantly increased in GAP-43^−/−^ cardiomyocytes in comparison with WT ones (Figure 2B). Moreover, we performed experiments comparing the peak amplitude of Ca^2+^ oscillations recorded in WT and GAP-43^−/−^ cardiomyocytes. The temporal analysis of the Ca^2+^ signal was analyzed using Clampfit software (ver. 10.2.0.18, Molecular Device, San Jose, CA, USA). The results revealed that the mean peak amplitude ± S.E.M. was 1.166 ± 0.008635 (N = 3, n = 522) and 1.324 ± 0.01280 (N = 3, n = 438) for WT and GAP-43^−/−^ cardiomyocyte traces, respectively (Student’s *t*-test *p* < 0.0001).

**Figure 3:**
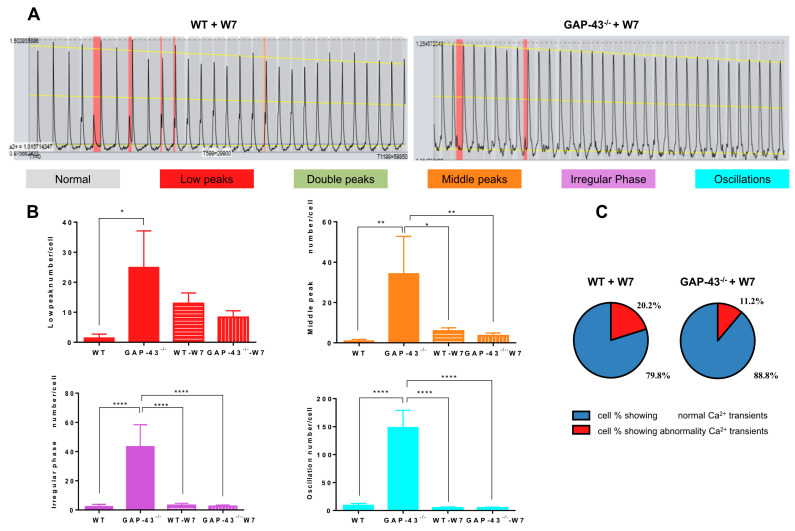
CaM inhibitor restored Ca^2+^ dyshomeostasis in GAP-43^−/−^ cardiomyocytes. (**A**) Representative Ca^2+^ signal abnormality patterns detected by AnomalyExplorer software [15] in WT (left image) and GAP-43^−/−^ (right image) cardiomyocytes loaded with Fluo-4 and treated with 20 μM W7, a CaM inhibitor. Each color-coded chart represents both normal Ca^2+^ signals (in gray) and different abnormalities such as low peaks, double peaks, medium peaks, oscillations, and irregular phases. (**B**) Quantitative analysis of the different Ca^2+^ transient abnormalities. The values are means ± S.E.M. from N = three biological samples; a total of 1589 WT+W7 and 1547 GAP-43^−/−^+W7 cardiomyocytes were examined (the data related to WT and GAP-43^−/−^ cardiomyocytes are those shown in Figure 2). * *p* < 0.05, ** *p* < 0.01, **** *p* < 0.0001 (ANOVA with post-hoc Tukey’s multiple comparison test). (**C**) Pie charts indicate the cell percentage showing different Ca^2+^ transient signals.

Interestingly, the treatment of cardiomyocytes with 20 µM W7, a CaM inhibitor, was able to drastically reduce the number of GAP-43^−/−^ cardiomyocytes showing abnormalities and the differences observed in the subgroups of recognized anomalies between WT and GAP-43^−/−^ cardiomyocytes in the same experimental conditions (Figure 3B,C).

**Figure 4:**
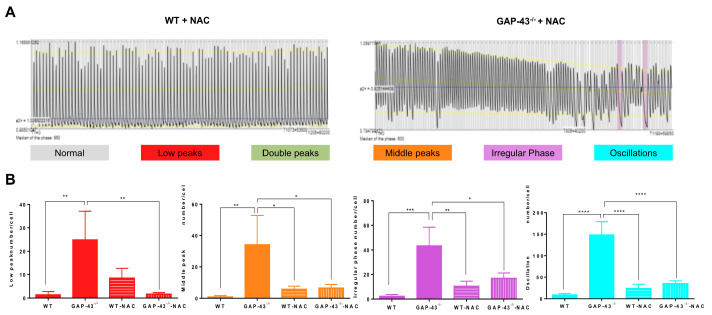
Impact of the antioxidant NAC on spontaneous Ca^2+^ oscillations in GAP-43^−/−^ cardiomyocytes. (**A**) Representative Ca^2+^ signal abnormality patterns detected by AnomalyExplorer software [15] in WT (left image) and GAP-43^−/−^ (right image) cardiomyocytes loaded with Fluo-4 and treated with 1 mM NAC, a pharmacological antioxidant. Each color-coded chart represents both normal Ca^2+^ signals (in gray) and different abnormalities such as low peaks, double peaks, medium peaks, oscillations, and irregular phases. (**B**) Quantitative analysis of the different Ca^2+^ transient abnormalities. The values are means ± S.E.M. from N = three biological samples, a total number of 739 WT+NAC and 681 GAP-43^−/−^ + NAC cardiomyocytes were examined (the data related to WT and GAP-43^−/−^ cardiomyocytes are those shown in Figure 2). * *p* < 0.05, ** *p* < 0.01, *** *p* < 0.001, **** *p* < 0.0001 (ANOVA with post-hoc Tukey’s multiple comparison test).

Also, the treatment of the GAP-43^−/−^ cardiomyocytes with 1 mM NAC, an antioxidant, reduced the alterations in the spontaneous Ca^2+^ variations, although to a lesser extent than the treatment with W7 (Figure 4).

### 3.3. Expression Levels of Ca^2+^ Handling Proteins in GAP-43^−/−^ Cardiomyocytes

**Figure 5:**
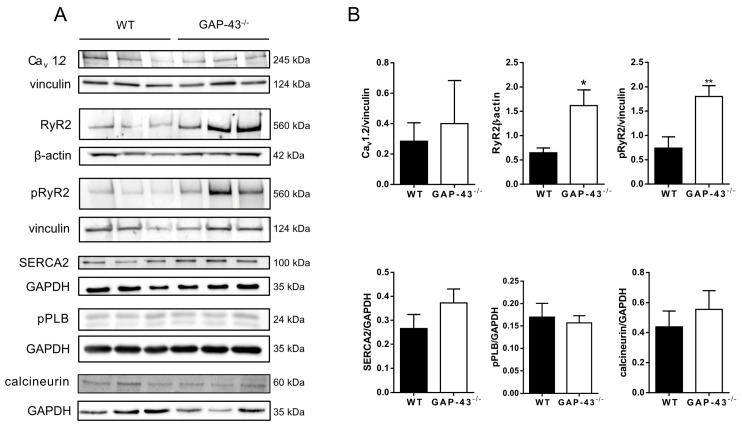
Expression levels of Ca^2+^ handling proteins. (**A**) Immunoblots of Cav 1.2, RyR2, pRyR2, SERCA2, pPLB, and calcineurin from WT or GAP-43^−/−^ cardiomyocytes. (**B**) Corresponding densitometric analyses of Western blotting in panel A. The densitometric analyses are plotted as the ratio between the optical density (OD) × mm^2^ of each band and the OD × mm^2^ of the corresponding loading control (vinculin, β-actin or GAPDH); Western blotting of pPLB was stripped and reprobed for GAPDH, and Western blotting of calcineurin was stripped and reprobed for GAPDH. Data are expressed as means ± S.E.M. from N= three biological samples. * *p* < 0.05, ** *p* < 0.01 (Student’s *t*-test).

Considering the importance of Ca^2+^ homeostasis in cardiomyocytes, we evaluated the expression levels of sarcolemma, sarcoplasmic reticulum, and cytosolic proteins involved in intracellular Ca^2+^ handling (Figure 5A).

In GAP-43^−/−^ cardiomyocytes, we observed a slight, although not significant, increase in the expression of the voltage-dependent Ca^2+^ channel Cav1.2, while the expression of ryanodine receptor type 2 (RyR2) and its phosphorylated form (pRyR2) was significantly increased (Figure 5B). The expression levels of the sarcoplasmic reticulum Ca^2+^-ATP pump type 2 (SERCA2), of the phosphorylated form of phospholamban (pPLB), an intracellular regulator of SERCA2, and of the calcineurin, a Ca^2+^-activated protein phosphatase involved in the regulation of many cardiac functions, did not change in GAP-43^−/−^ cardiomyocytes in respect to WT ones (Figure 5B).

### 3.4. GAP-43^−/−^ Cardiomyocytes Produce Increased Amounts of ROS

**Figure 6:**
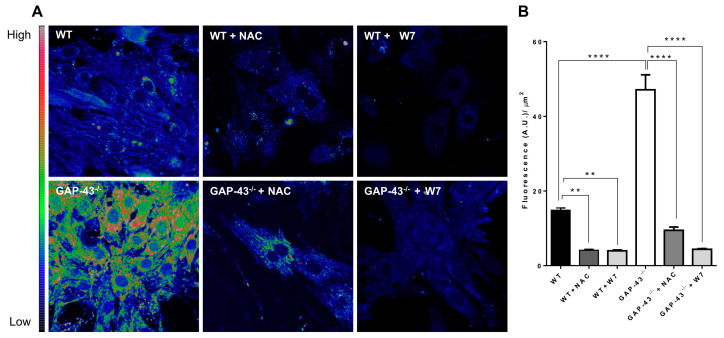
ROS levels in GAP-43^−/−^ cardiomyocytes. (**A**) Representative confocal images of WT or GAP-43^−/−^ cardiomyocytes, without or with 1 mM NAC or 20 µM W7, loaded with H_2_DCFDA. (**B**) Quantitative analysis of ROS levels in cardiomyocytes, expressed as a ratio between cell mean fluorescence intensity (Arbitrary Unity, A.U.) and cell area (µm^2^). The values are means ± S.E.M. from N = three biological samples; a total number of 375 WT, 329 WT + NAC, 440 WT + W7, 300 GAP-43^−/−^, 280 GAP-43^−/−^ + NAC, 429 GAP-43^−/−^ + W7 cardiomyocytes were examined. ** *p* < 0.01, **** *p* < 0.0001 (ANOVA with post-hoc Tukey’s multiple comparison test).

Given the crosstalk between Ca^2+^ homeostasis and oxidative stress, ROS levels were assayed using a specific dye (H2DCF-DA) and confocal microscopy. The results showed that ROS levels were about three-fold greater in GAP-43^−/−^ cardiomyocytes compared to WT ones (Figure 4A,B). As expected, the administration of the antioxidant NAC (1 mM) decreased ROS levels. In the presence of the specific inhibitor of CaM (20 µM W7), ROS levels were strongly reduced in GAP-43^−/−^ cardiomyocytes. To note, we also observed a significant reduction in ROS levels in WT cardiomyocytes treated with W7 or NAC (Figure 6A,B).

We investigated the expression levels of NOX2, one of the main cytoplasmic enzymes capable of generating ROS, and of the antioxidant enzymes SOD1, SOD2, and catalase. No significant differences were found between the tested genotypes (see Supplemental Material Figure S1: Expression of pro-oxidant and antioxidant enzymes).

### 3.5. GAP-43^−/−^ Mitochondria Show Altered Morphology and Metabolism

**Figure 7:**
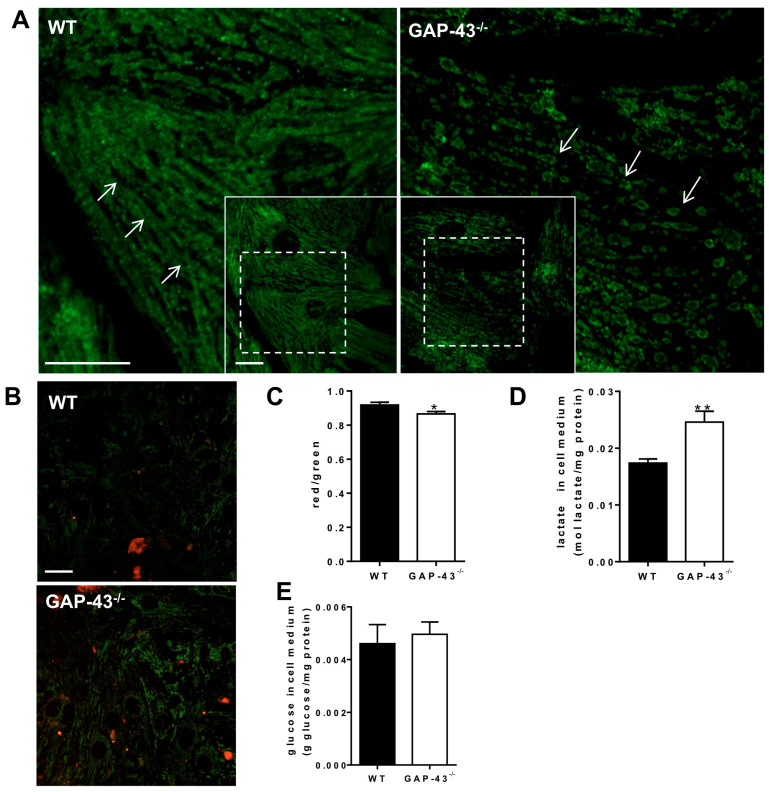
Mitochondria morphology and metabolism changes in GAP-43^−/−^ cardiomyocytes. (**A**) Representative high-magnification confocal images (corresponding to insert dotted-line boxes) of mitochondria in WT and GAP-43^−/−^ cardiomyocytes immunostained with anti-TOM20 (bar = 20 µm; the insert solid-line boxes show the low magnification acquired field). The white arrows indicate the different mitochondria morphology in WT and in GAP-43^−/−^ cardiomyocytes. (**B**) Representative confocal images of JC-1-stained mitochondria in WT and GAP-43^−/−^ cardiomyocytes (bar = 20 µm). (**C**) Mitochondrial membrane potential in WT and GAP-43^−/−^ cardiomyocytes loaded with JC-1, plotted as the ratio of red and green fluorescence of JC-1. Data are means ± S.E.M. from N = three biological samples; a total number of 523 WT and 681 GAP-43^−/−^ cardiomyocytes were examined. (**D**) Lactate released in cardiomyocyte cell medium. (**E**) Glucose levels in cardiomyocyte cell medium. Data in panels (**D**,**E**) are means ± S.E.M. from N = six biological samples of WT cardiomyocyte cultures and from five biological samples of GAP-43^−/−^ cardiomyocyte cultures. * *p* < 0.05; ** *p* < 0.01 (Student’s *t*-tests).

Considering the increased ROS levels in GAP-43^−/−^ cardiomyocytes, we evaluated the morphological aspects of mitochondria and analyzed features of mitochondrial functionality. Mitochondrial morphology was highlighted by the staining of outer membrane translocase 20 (TOM20), and images were acquired by confocal microscopy. The immunofluorescence staining depicted a higher proportion of elongated mitochondria in WT cardiomyocytes than in GAP-43^−/−^ ones, in which mitochondria appeared more spheroid- or donut-like shaped (Figure 7A). To verify whether the morphological alterations were also accompanied by differences in mitochondrial health, we used JC-1 to measure the degree of mitochondrial polarization. These experiments revealed that the mitochondrial membrane potential was reduced in GAP-43^−/−^ cardiomyocytes in comparison to WT ones (Figure 7B), indicating the presence of an unhealthy status. Furthermore, indirect evidences of an altered mitochondria metabolism in GAP-43^−/−^ cardiomyocytes came from increased levels of lactate without corresponding glucose consumption found in the culture medium of GAP-43^−/−^ cardiomyocytes (Figure 7C,D).

### 3.6. GAP-43^−/−^ Mitochondria Show Ca^2+^ Overload and Higher Superoxide Production

**Figure 8:**
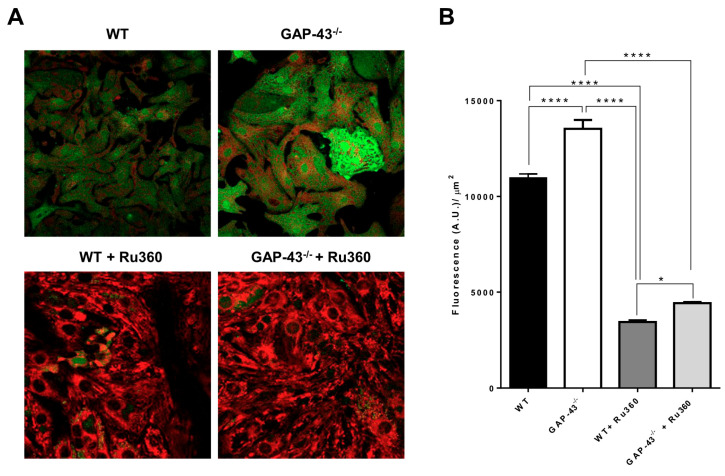
Increased mitochondrial Ca^2+^ levels in GAP-43^−/−^ cardiomyocytes. (**A**) Representative confocal images of WT and GAP-43^−/−^ cardiomyocytes monitored in the absence (top images) or presence (bottom images) of Ru360, a specific mitochondrial Ca^2+^ uptake inhibitor. Cells were loaded with Fluo-4 (green fluorescence) and Mitotracker Deep Red (red fluorescence) in order to measure mitochondrial Ca^2+^. (**B**) Quantitative analysis of mitochondrial Ca^2+^ levels calculated in cell regions in which Fluo-4 and Mito-Tracker Deep Red signals co-localized. For each cell, the mitochondrial Fluo-4 mean fluorescence intensity value (expressed as arbitrary unit, A.U.), and area were calculated; the data were expressed as mean fluorescence per area unit (A.U./µm^2^). The values are means ± S.E.M. from N = three biological samples; a total number of 739 WT, 415 GAP-43^−/−^, 551 WT+Ru360, 546 GAP-43^−/−^+Ru360 cardiomyocytes were examined. * *p* < 0.05, **** *p* < 0.0001 (ANOVA with post-hoc Tukey’s multiple comparison test).

To find the cause of the morphological and functional alterations in mitochondria, considering the results on Ca^2+^ homeostasis reported above, we analyzed the Ca^2+^ content in mitochondria by using confocal microscopy and two dyes, Fluo-4 and MitoTracker Deep Red, to highlight Ca^2+^ levels in the mitochondrial area. Compared to WT cardiomyocytes, the GAP-43^−/−^ ones showed an increase in mitochondrial Ca^2+^ levels, abolished by Ru360, a specific inhibitor of mitochondrial Ca^2+^ uniporter (MCU), which allows Ca^2+^ entry into mitochondria (Figure 8A,B).

**Figure 9:**
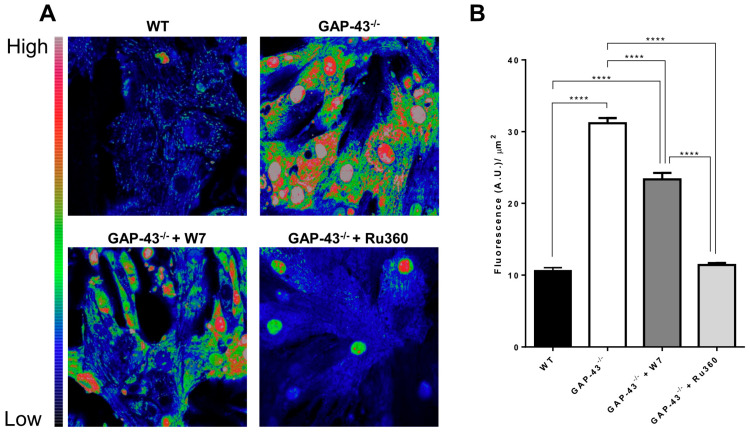
Increased superoxide anion levels in GAP-43^−/−^ cardiomyocytes. (**A**) Representative confocal images of WT cardiomyocytes and GAP-43^−/−^ cardiomyocytes without (top images) or with W7 or Ru360 (bottom left and bottom right images, respectively) loaded with MitoSOX, a mitochondrial superoxide anion indicator. (**B**) Quantitative analysis of O_2_^●−^ levels expressed as a ratio between cell mean fluorescence intensity (O_2_^●−^) and area unit (µm^2^). Data are means ± S.E.M. derived from N = three biological samples; a total number of 577 WT, 947 GAP-43^−/−^, 908 GAP-43^−/−^ + W7, 808 GAP-43^−/−^+Ru360 cardiomyocytes were examined. **** *p* < 0.0001 (ANOVA with post-hoc Tukey’s multiple comparison test).

The excessive mitochondrial oxidative stress is critically dependent on mitochondrial Ca^2+^ loading that promote the high production of ROS. We evaluated the levels of superoxide anion (O_2_^•−^) using MitoSOX and confocal microscopy. The results showed that O_2_^•−^ levels increased about three-fold in GAP-43^−/−^ cardiomyocytes’ mitochondria compared to WT ones (Figure 9A,B). The superoxide anion increase was only partially counteracted by treatment with W7, a CaM inhibitor, but the presence of Ru360, blocking mitochondrial Ca^2+^ uptake, abolished the excess superoxide anion generation observed in GAP-43^−/−^ mitochondria (Figure 9A,B).

## 4. Discussion

Since its discovery in rat synaptosomes more than 30 years ago, many results have shown that GAP-43 could also play a functional role in tissues other than nervous system tissues. GAP-43, thanks to its IQ domain, can bind CaM, modulating its availability to downstream targets in relationship to intracellular Ca^2+^ concentrations. Both past and recent results from our laboratory reveal that GAP-43 is expressed in skeletal and cardiac muscles [4,6,7,9]. In both tissues, it shares similar locations near the Ca^2+^ release units, and, interestingly, GAP-43^−/−^ mice develop cardiac hypertrophy [11]. Indeed, in mouse cardiomyocytes, GAP-43 is involved in the control of intracellular Ca^2+^ homeostasis and ROS balance that in turn, if altered, can be a cause of, or at least contribute to, cardiomyocyte hypertrophy. Hypertrophic stimuli activate a complex signaling cascade involving Ca^2+^-CaM signaling, but also MAPK and PI-3K [18], that converge onto a common program targeting the activity of specific transcription factors such as GATA-4 and Nkx-2.5 [19,20]. We found that the hypertrophic phenotype is maintained in in vitro isolated GAP-43^−/−^ cardiomyocytes, indicating that the hypertrophic program is probably induced by the lack of expression of GAP-43. Indeed, after dissociation and 7 days of culture, GAP-43^−/−^ cardiomyocytes, compared to WT, maintained higher expression levels of GATA-4 and Nkx-2.5; this would lead to the exclusion of external regulatory factors, such as overloading pressure, promoting the hypothesis that the absence of GAP-43 could activate a hypertrophy program. In this respect, in our experimental condition, we found that GAP-43^−/−^ cardiomyocytes expressed increased levels of cMyH and α-actinin, both markers of cardiac hypertrophy [21].

One emerging piece of evidence from our study is related to the disruption of Ca^2+^ homeostasis in GAP-43^−/−^ cardiomyocytes. In cardiac cells, Ca^2+^ has a central role in triggering contraction and relaxation processes, but it is also involved in mediating intracellular signaling directly or through the Ca^2+^-CaM complex [22]. Similar alterations in the pattern of spontaneous Ca^2+^ oscillations were also found in GAP-43^−/−^ myotubes, highlighting a common mechanism involving GAP-43 and CaM in the control of Ca^2+^ homeostasis in striated muscles [9]. This aspect is also confirmed by the evidence that W7, a CaM inhibitor, counteracted the Ca^2+^ alterations in GAP-43^−/−^ cardiomyocytes. Interestingly, GAP-43 localizes in the papillary muscle of adult mice close to α-actinin depicting spots at the two sides of the Z-line [11]. This localization could reflect the functional role of GAP-43 in the cardiac muscle; indeed, in a position close to the dyads, GAP-43 interacting with CaM could play a role in modulating the dyads’ Ca^2+^ release during the contraction/relaxation process. Due to its physiological role, alterations to Ca^2+^ homeostasis are known as a major contributor to heart dysfunction, as it plays a central role in systolic and diastolic changes, arrhythmogenesis, and hypertrophy [23,24]. In cardiac myocytes, systolic Ca^2+^ transient occurs when Ca^2+^ is released from the sarcoplasmic reticulum (SR). SR Ca^2+^ release is triggered by Ca^2+^ influx, through L-type Ca^2+^ channels involving RyR2 channels by the Ca^2+^-induced Ca^2+^ release (CICR) mechanism. In the analysis of the expression of the main proteins involved in intracellular Ca^2+^ signaling, we observed an increase in RyR2 and its phosphorylated form in GAP-43^−/−^ cardiomyocytes, while Cav 1.2 and SERCA2 were unchanged. These data suggest that the increased active phosphorylated CaMKII enhances RyR2 phosphorylation at Ser2815, which results in a more active RyR2 channel status [25]. Moreover, RyR2 phosphorylation influences dyad arrangement, increasing the probability of Ca^2+^ sparks linked to a probably positive allosteric effect on channel opening [26]. These modifications could modify physiological Ca2+ handling, unbalancing the ratio between Ca^2+^ release and storage, introducing abnormalities in CICR, often associated with cardiac hypertrophy [27,28]. In any case, these are only hypotheses derived from published evidence; the direct link between the increased expression of RyR2 and its phosphorylated form and altered CICR and hypertrophy needs to be explored through a new, detailed experimental plane in our model. Numerous studies have indicated the critical role of the CaM-RyR2 interaction in the fine regulation of SR Ca^2+^ release, where alterations in the interaction of these proteins can lead to the development of heart disease [29,30,31]. Alterations in intracellular Ca^2+^ homeostasis can also have an effect on CaMKII activity. In GAP-43^−/−^ cardiomyocytes, we found a significant increase in the active/phosphorylated form of CaMKII; this result is in line with evidence linking the frequency of Ca^2+^ oscillations to Ca/CaM elevations and CaMKII transition in the phosphorylated state [32]. Phosphorylated CaMKII is a key element in cardiomyocyte physiology due to its ability to regulate numerous signaling linked to excitation–contraction coupling and excitation–transcription coupling [33]. It is interesting to note that CaM and CaMKII are localized in many sites in the cardiomyocytes, including the nucleus; however, the highest concentrations appear at the transverse tubules where excitation–contraction coupling occurs and where we also found GAP-43 to be located [11,34]. So, in GAP-43^−/−^ cardiomyocytes, the loss of interaction between GAP-43 and CaM could be responsible for altered CaMKII activity. We found that GAP-43^−/−^ cardiomyocytes are more prone to producing ROS. The treatment of GAP-43^−/−^ cardiomyocytes with an antioxidant such as NAC reduces ROS production, although to a less significant extent compared to WT cultures. However, the use of W7, capable of inhibiting CaM-dependent activities, appears to be more effective in significantly reducing ROS levels in GAP-43^−/−^ cardiomyocytes. The ROS increase in GAP-43^−/−^ cardiomyocytes appears to be accompanied by an altered mitochondria morphology with the appearance of donut-shaped mitochondria indicative of an alteration in mitochondrial functionality. Indeed, the presence of a metabolic imbalance in these mitochondria is supported by a modest but significant reduction in mitochondrial membrane potential and an increase in lactate production, although glucose consumption does not appear to be altered. These findings are particularly significant since many studies suggest that a variation in the redox status in cardiomyocytes can cause, or contribute to, the development of a hypertrophic phenotype [35,36]. To investigate the relationship between the observed Ca^2+^ dyshomeostasis and ROS imbalance, we focused our attention on the role of the mitochondrial Ca^2+^ uniporter. The comparison of mitochondrial Ca^2+^ levels in GAP-43^−/−^ and WT cardiomyocytes highlighted a significant increase in mitochondrial Ca^2+^ in the former; this increase was blocked using Ru360, an MCU inhibitor. Increased mitochondrial Ca^2+^ levels via MCU observed in GAP-43^−/−^ cardiomyocytes correlates with what is known in the literature in relation to the interplay between mitochondrial respiration/production of free radicals and Ca^2+^ variations [37]. The major mitochondrial redox molecules are the superoxide anion radical (O_2_^•−^) and the hydrogen peroxide. Mainly, O_2_^•−^ is produced in the electron transfer chains in mitochondria generated by electrons that leak from complex I and III that reduce O_2_ [38]. Assaying superoxide anion levels, we found that the mitochondria of GAP-43^−/−^ cardiomyocytes showed a significant increase in superoxide anion levels, blunted by W7 but completely reversed by Ru360. Also, other authors have reported that a reduction in the expression levels of MCU by means of shRNA or its inhibition using Ru360 prevented mitochondrial Ca^2+^ overload in hypertrophic models [39]. From another perspective, it has been reported that mitochondrial Ca^2+^ accumulation by MCU enhancer promotes proarrhythmic spontaneous Ca^2+^ waves in rat ventricular myocytes that in turn had detrimental effects on intracellular Ca^2+^ handling and ROS production [40]. The MCU complex is finely regulated in the heart in physiological and pathological conditions (i.e., cardiac hypertrophy), implying that its function is dynamically regulated based on the context [41]. Other possible pathways should also be taken into consideration. In fact, GAP-43^−/−^ cardiomyocytes might exhibit changes in mitochondrial function. Ca^2+^ overload can lead to mitochondrial remodeling associated with a reduction in mitochondrial ATP production, changes in enzyme expression and activity, or both events [42,43]. Moreover, the alterations that we found could be responsible for the metabolic reprogramming of cardiomyocytes that in turn could influence the phenotype. As demonstrated in other models, lactate rich environment can modulate gene expression and modify the expression of proteins linked to cell hypertrophy and cytoskeleton remodeling [44,45]. In fact, GAP-43^−/−^ cells could rely more on glycolysis than oxidative phosphorylation. In the GAP-43^−/−^ model, we observed that both intracellular Ca^2+^ and ROS levels were imbalanced. The increased levels of ROS can lead to the oxidation of CaMKII that can also shift the activation of CaMKII from a Ca/CaM-dependent to -independent mode, favoring and prolonging its activation state. Indeed, it has been demonstrated that the Ca/CaM-independent activity of CaMKII is also a consequence of conditions that promote CaMKII oxidation [46]. CaMKII activation can potentially increase or downregulate myriad genes and the proteins that they encode. In fact, CaMKIIδ_C_ activates a cardiomyocyte apoptosis program by the way of the mitochondrial death pathway [47,48]; on the other hand, CaMKIIδ_B_ promotes cardiomyocyte survival and growth. This latter aspect appears to be promoted by CaMKII by means of two signaling pathways: the phosphorylation of HSF1 and HSP70 triggering [49] or the GATA-4-mediated co-activation and induction of the antiapoptotic protein Bcl-2 [50]. Even if we have no direct indication of the CaMKII isoform that is preferentially activated in our experiments, we found an increased expression of GATA-4 that is in line with the pro-survival/hypertrophy program activated in GAP-43^−/−^ cardiac tissue, as we previously demonstrated [11]. In this regard, preliminary experiments on the proteomic profile in WT and GAP-43^−/−^ cardiomyocytes suggest an inhibition of protein clusters involved in organ death and an activation of those promoting cell survival (unpublished preliminary data: manuscript in preparation). Furthermore, the loss of GAP-43 could dysregulate cytokine expression, leading to chronic low-grade inflammation, which is known to promote cardiac hypertrophy like NF-κB or JAK/STAT signaling [51,52]. These observations pave the way to further investigations by increasing the range of roles covered by GAP-43.

## 5. Limitations of the Study and Perspectives

In the present study, the use of a GAP-43^−/−^ model allowed us to investigate possible dysfunctions present in the cardiac muscle and to identify the role that GAP-43 plays in this tissue. On the other hand, GAP-43^−/−^ mice present a high neonatal mortality [1], and consequently a study on the cardiomyocytes of the adult heart is extremely difficult to conduct. For this reason, the present study was conducted on cardiomyocytes isolated from the hearts of newborn mice. However, it should be considered that neonatal cardiomyocytes do not fully recapitulate the characteristics of mature cardiac cells. Differences in Ca^2+^ handling, mitochondrial function, and hypertrophic signaling between neonatal and adult cardiomyocytes could affect the generalizability of the findings. However, the results obtained in neonatal GAP-43^−/−^ mice are compared with the corresponding WT at the same stages of development and maturation. This provides clear evidence of the possible functional role of the GAP-43 protein in cardiac homeostasis. For this reason, further validation in adult cardiomyocytes or in vivo models would strengthen our conclusions.

One limitation of this study is the lack of direct evidence establishing a causal link between increased intracellular Ca^2+^ levels, or else ROS levels, and the hypertrophic phenotype observed in GAP-43^−/−^ cardiac cells. Thus, even if the data suggest a correlation, further experiments are needed to determine whether and how increased Ca^2+^ levels, or increased ROS levels, directly contribute to hypertrophy or if additional signaling pathways are involved. The absence of GAP-43 could alter normal CaM signaling, resulting in increased Ca^2+^ levels with mitochondrial Ca^2+^ overload favoring an increase in ROS and the sustained activation of CaMKII, which could be the cause of, or contribute to, the activation of transcriptional signals leading to the observed hypertrophy. In addition, a more detailed investigation into mitochondrial morphology, ultrastructure, and metabolism is needed to better understand the proposed disruption of Ca^2+^ homeostasis.

In conclusion, the evidence that the absence of GAP-43 correlates with a hypertrophic phenotype and intracellular Ca^2+^ dyshomeostasis accompanied with increased ROS level open up new perspectives in this field of research, which will therefore require further investigation in the future.

## Data Availability

All of the data is contained within the article and Supplementary Materials.

## Supplementary Materials

The following supporting information can be downloaded at: https://www.mdpi.com/article/10.3390/antiox14030361/s1, Figure S1: Expression of pro-oxidant and antioxidant enzymes; Supplementary Method S1; Video S2: WT cardiomyocyte spontaneous Ca^2+^ oscillations; Video S3: GAP-43^−/−^ cardiomyocyte spontaneous Ca^2+^ oscillations.

## References

[1] Strittmatter S.M., Fankhauser C., Huang P.L., Mashimo H., Fishman M.C. (1995). Neuronal pathfinding is abnormal in mice lacking the neuronal growth cone protein GAP-43. Cell.

[2] da Cunha A., Vitkovic L. (1990). Regulation of immunoreactive GAP-43 expression in rat cortical macroglia is cell type specific. J. Cell Biol..

[3] Deloulme J.C., Janet T., Au D., Storm D.R., Sensenbrenner M., Baudier J. (1990). Neuromodulin (GAP43): A neuronal protein kinase C substrate is also present in 0-2A glial cell lineage. Characterization of neuromodulin in secondary cultures of oligodendrocytes and comparison with the neuronal antigen. J. Cell Biol..

[4] Stocker K.M., Ciment G., Baizer L. (1992). GAP-43 in non-neuronal cells of the embryonic chick limb: Clues to function. Perspect. Dev. Neurobiol..

[5] Heuss D., Schlotzer-Schrehardt U. (1998). Subcellular localization of phosphoprotein B-50 in regenerating muscle. An immuno-electron microscopic study. Neurol. Res..

[6] Guarnieri S., Morabito C., Paolini C., Boncompagni S., Pilla R., Fano-Illic G., Mariggio M.A. (2013). Growth associated protein 43 is expressed in skeletal muscle fibers and is localized in proximity of mitochondria and calcium release units. PLoS ONE.

[7] Caprara G.A., Perni S., Morabito C., Mariggio M.A., Guarnieri S. (2014). Specific association of growth-associated protein 43 with calcium release units in skeletal muscles of lower vertebrates. Eur. J. Histochem..

[8] Mosevitsky M.I. (2005). Nerve ending “signal” proteins GAP-43, MARCKS, and BASP1. Int. Rev. Cytol..

[9] Caprara G.A., Morabito C., Perni S., Navarra R., Guarnieri S., Mariggio M.A. (2016). Evidence for Altered Ca^2+^ Handling in Growth Associated Protein 43-Knockout Skeletal Muscle. Front. Physiol..

[10] Rahmati M., Taherabadi S.J. (2021). The effects of exercise training on Kinesin and GAP-43 expression in skeletal muscle fibers of STZ-induced diabetic rats. Sci. Rep..

[11] Bevere M., Morabito C., Guarnieri S., Mariggio M.A. (2022). Mice lacking growth-associated protein 43 develop cardiac remodeling and hypertrophy. Histochem. Cell Biol..

[12] Helms A.S., Alvarado F.J., Yob J., Tang V.T., Pagani F., Russell M.W., Valdivia H.H., Day S.M. (2016). Genotype-Dependent and -Independent Calcium Signaling Dysregulation in Human Hypertrophic Cardiomyopathy. Circulation.

[13] Turrens J.F. (2003). Mitochondrial formation of reactive oxygen species. J. Physiol..

[14] Aon M.A., Cortassa S., Marban E., O’Rourke B. (2003). Synchronized whole cell oscillations in mitochondrial metabolism triggered by a local release of reactive oxygen species in cardiac myocytes. J. Biol. Chem..

[15] Penttinen K., Siirtola H., Avalos-Salguero J., Vainio T., Juhola M., Aalto-Setala K. (2015). Novel Analysis Software for Detecting and Classifying Ca^2+^ Transient Abnormalities in Stem Cell-Derived Cardiomyocytes. PLoS ONE.

[16] Morabito C., Rovetta F., Bizzarri M., Mazzoleni G., Fano G., Mariggio M.A. (2010). Modulation of redox status and calcium handling by extremely low frequency electromagnetic fields in C2C12 muscle cells: A real-time, single-cell approach. Free Radic. Biol. Med..

[17] Berardini M., Gesualdi L., Morabito C., Ferranti F., Reale A., Zampieri M., Karpach K., Tinari A., Bertuccini L., Guarnieri S. (2023). Simulated Microgravity Exposure Induces Antioxidant Barrier Deregulation and Mitochondria Enlargement in TCam-2 Cell Spheroids. Cells.

[18] Tham Y.K., Bernardo B.C., Ooi J.Y., Weeks K.L., McMullen J.R. (2015). Pathophysiology of cardiac hypertrophy and heart failure: Signaling pathways and novel therapeutic targets. Arch. Toxicol..

[19] Liang Q., De Windt L.J., Witt S.A., Kimball T.R., Markham B.E., Molkentin J.D. (2001). The transcription factors GATA4 and GATA6 regulate cardiomyocyte hypertrophy in vitro and in vivo. J. Biol. Chem..

[20] Akazawa H., Komuro I. (2003). Roles of cardiac transcription factors in cardiac hypertrophy. Circ. Res..

[21] Ridinger H., Rutenberg C., Lutz D., Buness A., Petersen I., Amann K., Maercker C. (2009). Expression and tissue localization of beta-catenin, alpha-actinin and chondroitin sulfate proteoglycan 6 is modulated during rat and human left ventricular hypertrophy. Exp. Mol. Pathol..

[22] Sorensen A.B., Sondergaard M.T., Overgaard M.T. (2013). Calmodulin in a heartbeat. FEBS J..

[23] Bers D.M. (2006). Altered cardiac myocyte Ca regulation in heart failure. Physiology.

[24] Mazeto I.F.S., Okoshi K., Silveira C., Sant’Ana P.G., Silva V.L.D., Mota G.A.F., Souza S.L.B., Vileigas D.F., Padovani C.R., Cicogna A.C. (2021). Calcium homeostasis behavior and cardiac function on left ventricular remodeling by pressure overload. Braz. J. Med. Biol. Res..

[25] Wehrens X.H., Lehnart S.E., Reiken S.R., Marks A.R. (2004). Ca^2+^/calmodulin-dependent protein kinase II phosphorylation regulates the cardiac ryanodine receptor. Circ. Res..

[26] Asghari P., Scriven D.R., Ng M., Panwar P., Chou K.C., van Petegem F., Moore E.D. (2020). Cardiac ryanodine receptor distribution is dynamic and changed by auxiliary proteins and post-translational modification. eLife.

[27] Yano M., Ikeda Y., Matsuzaki M. (2005). Altered intracellular Ca^2+^ handling in heart failure. J. Clin. Investig..

[28] Muth J.N., Bodi I., Lewis W., Varadi G., Schwartz A. (2001). A Ca^2+^-dependent transgenic model of cardiac hypertrophy: A role for protein kinase Calpha. Circulation.

[29] Yamaguchi N., Takahashi N., Xu L., Smithies O., Meissner G. (2007). Early cardiac hypertrophy in mice with impaired calmodulin regulation of cardiac muscle Ca release channel. J. Clin. Investig..

[30] Xu X., Yano M., Uchinoumi H., Hino A., Suetomi T., Ono M., Tateishi H., Oda T., Okuda S., Doi M. (2010). Defective calmodulin binding to the cardiac ryanodine receptor plays a key role in CPVT-associated channel dysfunction. Biochem. Biophys. Res. Commun..

[31] Ono M., Yano M., Hino A., Suetomi T., Xu X., Susa T., Uchinoumi H., Tateishi H., Oda T., Okuda S. (2010). Dissociation of calmodulin from cardiac ryanodine receptor causes aberrant Ca^2+^ release in heart failure. Cardiovasc. Res..

[32] De Koninck P., Schulman H. (1998). Sensitivity of CaM kinase II to the frequency of Ca^2+^ oscillations. Science.

[33] Bers D.M. (2011). Ca^2+^-calmodulin-dependent protein kinase II regulation of cardiac excitation-transcription coupling. Heart Rhythm..

[34] Bers D.M., Grandi E. (2009). Calcium/calmodulin-dependent kinase II regulation of cardiac ion channels. J. Cardiovasc. Pharmacol..

[35] Takimoto E., Kass D.A. (2007). Role of oxidative stress in cardiac hypertrophy and remodeling. Hypertension.

[36] Sabri A., Hughie H.H., Lucchesi P.A. (2003). Regulation of hypertrophic and apoptotic signaling pathways by reactive oxygen species in cardiac myocytes. Antioxid. Redox Signal.

[37] Pitter J.G., Maechler P., Wollheim C.B., Spat A. (2002). Mitochondria respond to Ca2+ already in the submicromolar range: Correlation with redox state. Cell Calcium.

[38] Kuznetsov A.V., Margreiter R., Ausserlechner M.J., Hagenbuchner J. (2022). The Complex Interplay between Mitochondria, ROS and Entire Cellular Metabolism. Antioxidants.

[39] Alves-Figueiredo H., Silva-Platas C., Estrada M., Oropeza-Almazan Y., Ramos-Gonzalez M., Bernal-Ramirez J., Vazquez-Garza E., Tellez A., Salazar-Ramirez F., Mendez-Fernandez A. (2024). Mitochondrial Ca^2+^ Uniporter-Dependent Energetic Dysfunction Drives Hypertrophy in Heart Failure. JACC Basic Transl. Sci..

[40] Hamilton S., Terentyeva R., Kim T.Y., Bronk P., Clements R.T., O-Uchi J., Csordas G., Choi B.R., Terentyev D. (2018). Pharmacological Modulation of Mitochondrial Ca^2+^ Content Regulates Sarcoplasmic Reticulum Ca^2+^ Release via Oxidation of the Ryanodine Receptor by Mitochondria-Derived Reactive Oxygen Species. Front. Physiol..

[41] Zaglia T., Ceriotti P., Campo A., Borile G., Armani A., Carullo P., Prando V., Coppini R., Vida V., Stolen T.O. (2017). Content of mitochondrial calcium uniporter (MCU) in cardiomyocytes is regulated by microRNA-1 in physiologic and pathologic hypertrophy. Proc. Natl. Acad. Sci. USA.

[42] Malyala S., Zhang Y., Strubbe J.O., Bazil J.N. (2019). Calcium phosphate precipitation inhibits mitochondrial energy metabolism. PLoS Comput. Biol..

[43] Walkon L.L., Strubbe-Rivera J.O., Bazil J.N. (2022). Calcium Overload and Mitochondrial Metabolism. Biomolecules.

[44] Ordono J., Perez-Amodio S., Ball K., Aguirre A., Engel E. (2022). The generation of a lactate-rich environment stimulates cell cycle progression and modulates gene expression on neonatal and hiPSC-derived cardiomyocytes. Biomater. Adv..

[45] Luti S., Militello R., Pinto G., Illiano A., Marzocchini R., Santi A., Becatti M., Amoresano A., Gamberi T., Pellegrino A. (2024). Chronic lactate exposure promotes cardiomyocyte cytoskeleton remodelling. Heliyon.

[46] Erickson J.R., Joiner M.L., Guan X., Kutschke W., Yang J., Oddis C.V., Bartlett R.K., Lowe J.S., O’Donnell S.E., Aykin-Burns N. (2008). A dynamic pathway for calcium-independent activation of CaMKII by methionine oxidation. Cell.

[47] Zhu W.Z., Wang S.Q., Chakir K., Yang D., Zhang T., Brown J.H., Devic E., Kobilka B.K., Cheng H., Xiao R.P. (2003). Linkage of beta1-adrenergic stimulation to apoptotic heart cell death through protein kinase A-independent activation of Ca^2+^/calmodulin kinase II. J. Clin. Investig..

[48] Zhu W., Woo A.Y., Yang D., Cheng H., Crow M.T., Xiao R.P. (2007). Activation of CaMKIIdeltaC is a common intermediate of diverse death stimuli-induced heart muscle cell apoptosis. J. Biol. Chem..

[49] Peng W., Zhang Y., Zheng M., Cheng H., Zhu W., Cao C.M., Xiao R.P. (2010). Cardioprotection by CaMKII-deltaB is mediated by phosphorylation of heat shock factor 1 and subsequent expression of inducible heat shock protein 70. Circ. Res..

[50] Little G.H., Saw A., Bai Y., Dow J., Marjoram P., Simkhovich B., Leeka J., Kedes L., Kloner R.A., Poizat C. (2009). Critical role of nuclear calcium/calmodulin-dependent protein kinase IIdeltaB in cardiomyocyte survival in cardiomyopathy. J. Biol. Chem..

[51] Purcell N.H., Tang G., Yu C., Mercurio F., DiDonato J.A., Lin A. (2001). Activation of NF-kappa B is required for hypertrophic growth of primary rat neonatal ventricular cardiomyocytes. Proc. Natl. Acad. Sci. USA.

[52] Wagner M.A., Siddiqui M.A. (2012). The JAK-STAT pathway in hypertrophic stress signaling and genomic stress response. JAKSTAT.

