# Supplementary material for: Growth-Associated Protein-43 Loss Promotes Ca2+ and ROS Imbalance in Cardiomyocytes
Source: Antioxidants (Basel). 2025 Mar 19;14(3):361. doi: 10.3390/antiox14030361 (PMC11939155; doi:10.3390/antiox14030361)
Supplement: Supplementary file 1 [file antioxidants-14-00361-s001.zip › Bevere at al Supplementary Method S1.pdf]

Supplementary Method S1

Temporal analysis of Ca^{2+} transient was calculated as f/f0, where f is the mean fluorescence intensity signal of a selected cell area of a single loaded cell acquired during a time lapse, and f0 is the mean fluorescence intensity of the same cell calculated from the first time point acquired.
The Ca^{2+} transient temporal analysis calculated as f/f0 was subjected to AnomalyExplorer software (Penttinen et al. PLoS One. 2015, https://github.com/siirtola/AnomalyExplorer) for possible anomalies detection. Analyzing Ca^{2+} transient, the software detects abnormality subgroups: low, middle, oscillating and double peak anomalies and irregular phase abnormality as described in the paper and reported as follow:

- **low peak** is flagged if the following conditions are met:
1) the transient minimum value is within 15% of the local regression height from the local bottom regression line;
2) the transient maximum value is between user-defined percentage limits of the local regression height;
3) the previous and the following transient do not contain double peak anomalies. Once the ascending section is classified as a low peak, the following descending section is also flagged as a low peak if its minimum value is within ±10% of the current section's minimum, i.e., the peak is fairly symmetrical;
- **middle peak** is flagged if the following conditions are met:
1) the section height is within the user-defined percentage limits of the local regression height;
2) the transient maximum value is within 60% of the local middle regression line;
3) the transient minimum value is within 40% of the local middle regression line;
4) the section height is less than 75% of the height of the surrounding sections;
5) there is no low peak anomaly in the previous or this section.
- the anomalous **oscillation** is reported by comparing the transient heights and lengths in the analyzed section with the consecutive transients that are reduced below the defined percentage limit are reported as oscillating
- **double peak** is flagged if the following conditions are met:
1) the section maximum value is within 40% of the section height of the local top regression line;
2) the height of the section is within user-defined percentage limit of the local regression height;
3) the upper percentage limit determines how deep the double peak can be (where 100% is the local bottom regression line and 50% is the local middle regression line);
4) the height of the section is within ±25% of the height of the following section
- i**rregular phase** is flagged if the distance of peaks differs by a user-defined from the median of the peak distances. Only those peaks that do not exhibit any anomalies are considered, except the peaks that exhibit double peaks anomaly which are treated as a single peak that has the mean of the positions of double peaks as its reference value.
-**plateau abnormality** is flagged if the transient changes its rate of ascent or descent more than a user-defined percentage (for example 75%) within a section.

According to the software user interface, the following user-defined setting was used for all conditions analyzed: low peak (10-40%); medium peak (0-38%); double peak (20-28%); irregular phase (100%); plateau abnormality (75%); oscillation (31%).
