# Supplementary figures and images for: Growth-Associated Protein-43 Loss Promotes Ca2+ and ROS Imbalance in Cardiomyocytes
Source: Antioxidants (Basel). 2025 Mar 19;14(3):361. doi: 10.3390/antiox14030361 (PMC11939155; doi:10.3390/antiox14030361)

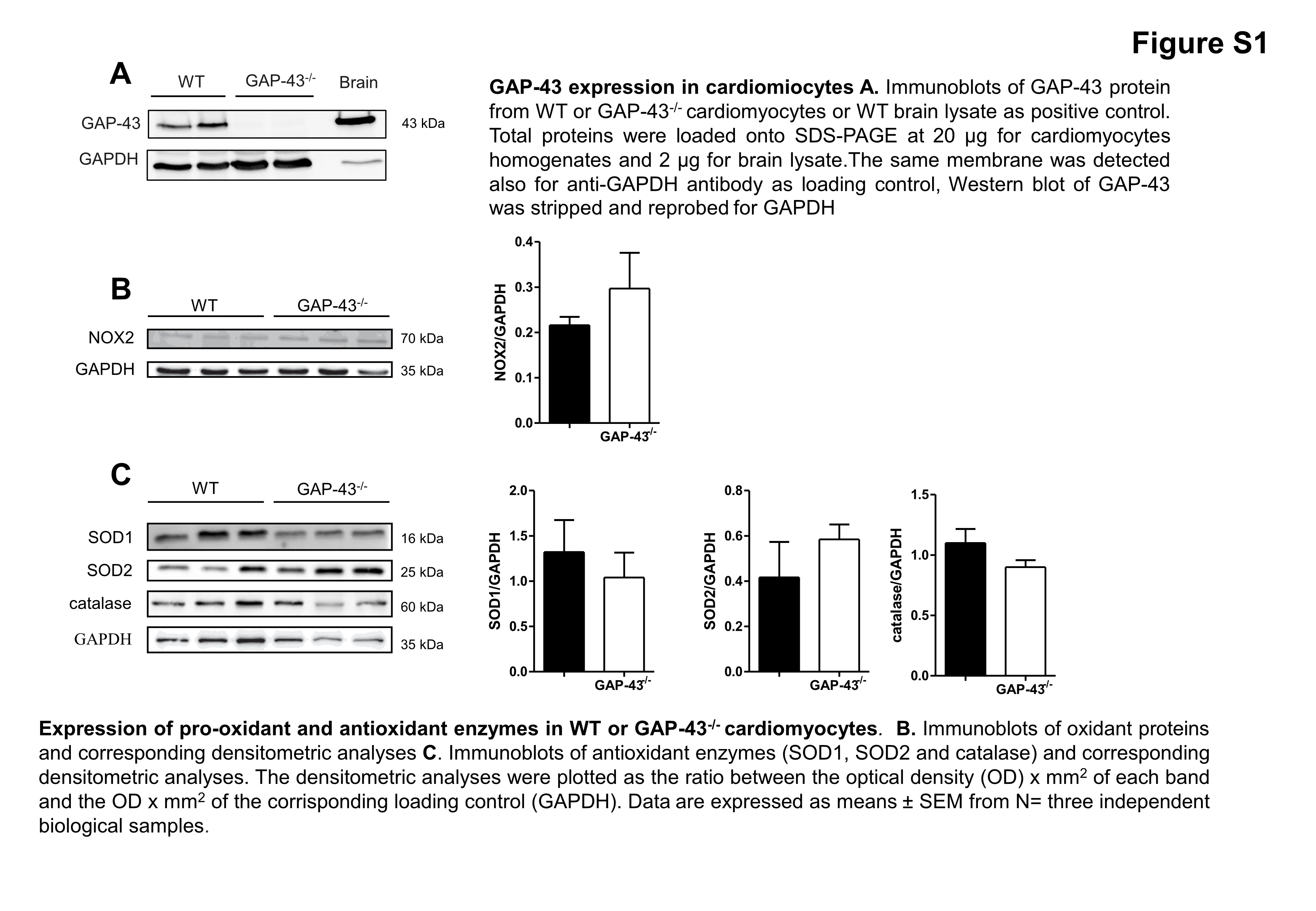

Supplement: Supplementary file 1 [file antioxidants-14-00361-s001.zip › Bevere et al Figure S1 Supplentary material.tiff]
